# Serial circulating tumor DNA to predict early recurrence in patients with hepatocellular carcinoma: a prospective study

**DOI:** 10.1002/1878-0261.13105

**Published:** 2021-10-04

**Authors:** Gui‐Qi Zhu, Wei‐Ren Liu, Zheng Tang, Wei‐Feng Qu, Yuan Fang, Xi‐Fei Jiang, Shu‐Shu Song, Han Wang, Chen‐Yang Tao, Pei‐Yun Zhou, Run Huang, Jun Gao, Hai‐Xiang Sun, Zhen‐Bin Ding, Yuan‐Fei Peng, Zhi Dai, Jian Zhou, Jia Fan, Ying‐Hong Shi

**Affiliations:** ^1^ Department of Liver Surgery and Transplantation Liver Cancer Institute Zhongshan Hospital Fudan University Shanghai China; ^2^ Key Laboratory of Carcinogenesis and Cancer Invasion of Ministry of Education Shanghai China; ^3^ Institutes of Biomedical Sciences Fudan University Shanghai China; ^4^ Shanghai Key Laboratory of Organ Transplantation Shanghai China; ^5^ State Key Laboratory of Genetic Engineering and Collaborative Innovation Center for Genetics and Development School of Life Sciences Fudan University Shanghai China

**Keywords:** biomarker, ctDNA, hepatocellular carcinoma, tumor recurrence

## Abstract

We studied the value of circulating tumor DNA (ctDNA) in predicting early postoperative tumor recurrence and monitoring tumor burden in patients with hepatocellular carcinoma (HCC). Plasma‐free DNA, germline DNA, and tissue DNA were isolated from 41 patients with HCC. Serial ctDNAs were analyzed by next‐generation sequencing before and after operation. Whole‐exome sequencing was used to detect the DNA of HCC and adjacent tissues. In total, 47 gene mutations were identified in the ctDNA of the 41 patients analyzed before surgery. ctDNA was detected in 63.4% and 46% of the patient plasma pre‐ and postoperation, respectively. The preoperative ctDNA positivity rate was significantly lower in the nonrecurrence group than in the recurrence group. With a median follow‐up of 17.7 months, nine patients (22%) experienced tumor recurrence. ctDNA positivity at two time‐points was associated with significantly shorter recurrence‐free survival (RFS). Tumors with *NRAS*, *NEF2L2*, and *MET* mutations had significantly shorter times to recurrence than those without mutations and showed high recurrence prediction performance by machine learning. Multivariate analyses showed that the median variant allele frequency (VAF) of mutations in preoperative ctDNA was a strong independent predictor of RFS. ctDNA is a real‐time monitoring indicator that can accurately reflect tumor burden. The median VAF of baseline ctDNA is a strong independent predictor of RFS in individuals with HCC.

## Graphical abstract

Here, we studied the value of circulating tumor DNA (ctDNA), in combination with germline and tissue DNA by whole‐exome sequencing in patients withhepatocellular carcinoma (HCC). Our analysis demonstrated that ctDNA may serve as a real‐time monitoring indicator by accurately reflecting tumor burden, and as a strong independent predictor of recurrence‐free survival in HCC.

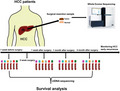

**Abbreviations**
AFP: α‐fetoprotein
ALT: alanine aminotransferase
BCLC: Barcelona Clinic Liver Cancer
cfDNA: circulating cell‐free DNA
CI: confidence interval
CNLC: China Liver Cancer Staging
ctDNA: circulating tumor DNA
GGT: γ‐glutamyl transpeptidase
HCC: hepatocellular carcinoma
HCV: hepatitis C virus
HR: hazard risk
MVI: microvascular invasion
NGS: next‐generation sequencing
RFS: recurrence‐free survival
ROC: receiver operating characteristic curve
TTR: time to recurrence
VAF: variant allele frequency

## 1 Introduction

Hepatocellular carcinoma (HCC) is the sixth most common cancer worldwide and the third common cause of patient death [1, 2, 3]. In spite of the development of many therapeutic treatments, liver resection and transplantation are the most common curative regimes [1, 2]. The shortage of liver donors and strict criteria of liver transplantation limit the application of liver transplantation, rendering liver resection the first‐line therapy for HCC [2]. However, the high incidence of HCC recurrence still confers a major challenge in the treatment of HCC [1, 2, 4].

Hence, how to monitor the tumor recurrence or progression effectively for HCC patients with liver resection is essential for the improvement of patient survival time [2]. Some researchers have identified several genetic predictors for HCC patient outcomes, including some somatic mutations in TGF‐β and Wnt signaling pathways, while others have reported some gene signatures predicting patient survival [4, 5, 6, 7]. However, these researches all use the tumor tissue, a notable limited practical use in our clinic practice, because the diagnosis of HCC is mainly according to CT/MRI criteria, and also only a small number of HCC patients need biopsy for pathological diagnosis [4]. In addition, tumor biopsy is limited by intratumoral heterogeneity and is also invasive, which limited the longitudinal evaluation of genetic variants in HCC [4].

Both healthy and malignant cells will release circulating cell‐free DNA (cfDNA) through tissue apoptosis and necrosis into the blood [4, 5, 8], which increased significantly in the inflammatory states, especial for malignancy [4, 5]. cfDNA levels can discriminate the malignancy from benign diseases such as colorectal cancer when coexisted with the inflammatory bowel disease [4, 5, 9, 10]. Specifically, tumor‐derived cfDNA (ctDNA) could be differentiated from wild‐type cfDNA by the identification of somatic variants in the tumor but not in the matched HCC tissue. Recently, many studies showed both nontumor cfDNA and ctDNA levels and the existence of genetic alterations in ctDNA are potential cancer biomarkers [5, 9, 10, 11]. The vital merit of ctDNA is that it confers dynamic detailed information about tumor biology whereas without the necessary for frequent biopsies [9, 10, 11, 12].

Recently, more and more researches have revealed that both non‐cancer‐specific plasma cfDNA and tumor‐derived plasma ctDNA showed remarkable prognostic prediction performance before treatment and for monitoring regime response in different cancers, including ovarian, breast, and lung cancers [5, 9, 10, 11, 12]. Serial ctDNA has been considered to be the most positive prognostic biomarker for monitoring the HCC patient survival benefits [13]. With the advanced bioinformatics technology, such as whole‐genome sequencing and next‐generation sequencing (NGS), processing data from cfDNA information could confer important genetic details on tumor phenotypes [9, 10, 11, 13, 14]. Even though ctDNA was proved to be a positive predictor after surgery for long‐term survival in HCC patients, there is still failure of evidence to verify its predictive value early on in tumor recurrence, prior to surgical resection [9, 11, 14]. Moreover, whether genomic characteristics and baseline ctDNA status before surgery could predict tumor sensitivity for liver resection still remains unclear. Therefore, we showed the yields of our prospective clinical trial that explore the value of HCC tumor tissue NGS and serial ctDNA analyses in the radical surgery therapy of HCC. We first described changes between preoperative and postoperative cfDNA levels or mutations in patients with HCC and then explored whether some known tumorigenic driver mutations commonly found in HCC tissue were still present in plasma ctDNA. Through the targeted NGS with largest panel of genes, we achieved the most comprehensive mutational profiles on the status quo from patient biopsies and cfDNA. Additionally, by monitoring prospectively clinicopathologic characteristics, our results provide the first testimony showing that ctDNA detection with genetic mutations could demonstrate vital knowledge on tumor recurrence prior to surgical resection. Issues including the potential of utilizing the tumors' genomic characteristics to predict early recurrence before HCC therapy and the value of ctDNA in dynamic surveillance of the disease were also explored. These results can contribute to predicting early recurrence and refining appropriate treatment approaches in advance.

## 2 Materials and methods

### 2.1 Study design and participants

In our prospective study, from May 2018 to December 2019, we recruited a total of 41 patients (all over 18 years of age) with confirmed radiological diagnosis [2] from Zhongshan Hospital, Fudan University. All those patients provided written informed consent in this study. Table 1 summarizes the clinical characteristics of this HCC cohort. Liver resection was performed on all enrolled 41 patients. After liver curative resection, all HCC patients were monitored regularly by analyses of tumor markers [α‐fetoprotein (AFP)], liver function tests, and abdominal ultrasound every 2 months. Also, if tumor recurrence was suspected, further CT or MRI scans were performed. Time to recurrence (TTR) was calculated from the date of liver curative surgery to the date of the diagnosis of recurrence. We also calculated recurrence‐free survival (RFS) from the date of surgical resection to the date of tumor recurrence or death. This study was approved by the institutional ethics committee of Zhongshan Hospital, Fudan University (B2019‐157R), and was performed in accordance with the 1975 Declaration of Helsinki [1, 2].

**Table 1:** Distribution of clinical variables among HCC patients (*n* = 41).

	No. of patients (%)
Sex	
Female	6 (14.6%)
Male	35 (85.4%)
Age	
< 50	7 (17.1%)
> 50	34 (82.9%)
HBsAg	
No	11 (26.8%)
Yes	30 (73.2%)
Anti‐HCV	
No	38 (92.7%)
Yes	3 (7.3%)
ALT (U·L^−1^)	
< 45	37 (90.2%)
> 45	4 (9.8%)
AFP (U·L^−1^)	
< 20	26 (63.4%)
> 20	15 (36.6%)
GGT (U·L^−1^)	
< 45	19 (46.3%)
> 45	22 (53.7%)
Liver cirrhosis	
No	12 (29.3%)
Yes	29 (70.7%)
Tumor number	
Single	32 (78.0%)
Multiple	9 (22.0%)
Tumor size (cm)	
< 5	18 (43.9%)
> 5	23 (56.1%)
Tumor capsule	
No	24 (58.5%)
Partial	10 (24.4%)
Complete	7 (17.1%)
MVI	
No	19 (46.3%)
Yes	22 (53.7%)
Tumor differentiation	
I–II	20 (48.8%)
II–III	21 (51.2%)
Adjuvant treatment	
No	13 (31.7%)
Yes	28 (68.3%)
BCLC	
0‐A	31 (75.6%)
B	10 (24.4%)
CNLC	
Ia‐Ib	33 (80.5%)
IIa‐IIb	8 (19.5%)

ALT, alanine aminotransferase; GGT, γ‐glutamyl transpeptidase; HBV, hepatitis B virus; HCV, hepatitis C virus.

### 2.2 Blood and HCC tissue sample collection

We collected peripheral blood samples from each patient in Cell‐Free DNA Collection Tubes (Roche, Basel, Switzerland) before hepatectomy. cfDNA was extracted from 4 mL of plasma within 1 week preoperatively and postoperatively (at 1 week, 1 month, and 4 months, respectively) using the AVENIO cfDNA Isolation Kit (Roche). Fresh tumor specimens were gained from HCC patients based on a 7‐point baseline sample collection protocol during surgery [15]. DNA was extracted from the tissues using the All Prep DNA/RNA Mini Kit (Qiagen, Dusseldorf, Germany) according to the manufacturer's instructions.

### 2.3 Next‐generation sequencing and variant calling

cfDNA was sequenced using the AVENIO ctDNA Surveillance Kit (Roche) to assess somatic mutations in 197 cancer‐related genes. The deduced sequencing depth for cfDNA ranged from 2000× to 10 000×. Variants were called with AVENIO ctDNA Analysis Software, which incorporates bioinformatics methods from CAPP‐Seq2 and iDES3 (integrated digital error suppression) to remove PCR duplicates and stereotypical errors from technical artifacts. ctDNA positivity was defined as when at least one mutation had been detected in matched ctDNA. Somatic variants were derived after filtering out common germline variants in public databases such as ExAC, dbSNP, and 1000 Genomes [16, 17], as well as variants detected in matched paracancerous tissue. Only nonsynonymous single‐nucleotide variants (SNVs) and insertions/deletions InDels) with an allele frequency (AF) of at least 0.1% were included in further analyses. The 1% cutoff value of variant allele frequency (VAF) was then prespecified for further outcome analyses according to previous study [18].

Whole‐exome sequencing was performed for each HCC and paracancerous tissue DNA using the TruSeq Exome Kit (Illumina, San Diego, CA, USA) according to the manufacturer’s instructions. The obtained exome libraries were paired‐end‐sequenced on the Illumina HiSeq X10 (2 × 150 bp) platform (Illumina). Trimmomatic V0.36 was used to trim bad reads or bases from raw data. The output clean data were then put into the sentieon DNAseq pipeline for read alignment, sample metric collection, duplicate read removal, indel realignment, and base quality score recalibration. The output recal.bam was then used as input for Sentieon TNscope to perform variant calling using matched paracancerous tissue as control. The unique average sequencing coverage ranged from 95× to 257× for tumors and from 105× to 252× for paracancerous tissues.

### 2.4 Statistical analysis

We described clinical variables using the mean ± standard deviation or median [interquartile range (IQR)] for distribution. Some correlations between ctDNA levels and clinical parameters were evaluated by the Wilcoxon rank‐sum test or the Kruskal–Wallis test. For all analyses, *P* < 0.05 means statistical significance. The Kaplan–Meier analysis was performed to assess the predictive value of AFP, microvascular invasion (MVI), and ctDNA, for example, in estimating RFS preoperatively by adjusting Barcelona Clinic Liver Cancer (BCLC) staging. Machine learning was applied to explore the prediction performance of preoperative ctDNA and clinical parameters with RFS based on R package ‘XGBoost or CoxBoost’ [19]. We carried out a multivariate Cox regression analysis to explore whether the variable parameters were associated with RFS. All analyses were carried out by using IBM spss (version 23.0, Chicago, MI, USA), r Statistics version 3.3.2 (R Foundation for Statistical Computing, Vienna, Austria) and graphpad prism (version 6.01, San Diego, CA, USA) software.

## 3 Results

### 3.1 Patient demographics and clinicopathologic characteristics

Information on patient clinicopathologic characteristics, and serial ctDNA in the surgical treatment are summarized in Tables 1 and 2. The mean age of the HCC patients was 73 years, and the majority were male (85.4%). Regarding the baseline clinical BCLC stage of patients, 31 patients (95.2%) had BCLC stage 0‐A and 10 patients (24.4%) had BCLC stage B disease; similarly, for China liver cancer staging (CNLC) stage, 33 patients (80.5%) had CNLC stage Ia/Ib and eight (19.5%) had CNLC stage IIa/IIb disease [20]. A total of 73.2% of patients had hepatitis B. At study entry, 15 (36.6%) patients had increased AFP levels, and 22 (53.7%) patients suffered from MVI. Pathology test demonstrated differentiated poorly tumors in 48.8% (20/41) of cases, with the rest being differentiated well to moderately. All patients underwent radical liver resection when diagnosed with HCC, with no perioperative mortality. With a median follow‐up of 17.7 months (range, 2.1–19.3), 13 patients (31.7%) had disease progression, among whom nine (Table S1) were found to have early recurrence (all patients suffered intrahepatic recurrence and all TTR < 2 years) after surgery and the other four patients died by the end of the follow‐up period.

**Table 2:** Clinicopathologic characteristics and tumor early recurrence as per ctDNA positivity.

Variables	Preoperative ctDNA		*P* value	Postoperative ctDNA		*P* value
	Negative	Positive		Negative	Positive	
Sex						
Female	3 (20.0%)	3 (11.5%)	0.460	4 (18.2%)	2 (10.5%)	0.489
Male	12 (80.0%)	23 (88.5%)		18 (81.8%)	17 (89.5%)	
Age (years)						
< 50	3 (20.0%)	4 (15.4%)	0.705	3 (13.6%)	4 (21.1%)	0.529
≥ 50	12 (80.0%)	22 (84.6%)		19 (86.4%)	15 (78.9%)	
HBsAg						
Negative	5 (33.3%)	6 (23.1%)	0.475	6 (27.3%)	5 (26.3%)	0.945
Positive	10 (66.7%)	20 (76.9%)		16 (72.7%)	14 (73.7%)	
Anti‐HCV treatment						
No	13 (86.7%)	25 (96.2%)	0.261	20 (90.9%)	18 (94.7%)	0.639
Yes	2 (13.3%)	1 (3.8%)		2 (9.1%)	1 (5.3%)	
ALT (U·L^−1^)						
< 75	12 (80.0%)	25 (96.2%)	0.093	19 (86.4%)	18 (94.7%)	0.368
≥ 75	3 (20.0%)	1 (3.8%)		3 (13.6%)	1 (5.3%)	
AFP (ng·mL^−1^)						
< 20	12 (80.0%)	14 (53.8%)	0.094	14 (63.6%)	12 (63.2%)	0.975
≥ 20	3 (20.0%)	12 (46.2%)		8 (36.4%)	7 (36.8%)	
GGT (U·L^−1^)						
< 54	8 (53.3%)	11 (42.3%)	0.495	12 (54.5%)	7 (36.8%)	0.257
≥ 54	7 (46.7%)	15 (57.7%)		10 (45.5%)	12 (63.2%)	
Liver cirrhosis						
No	5 (33.3%)	7 (26.9%)	0.664	6 (27.3%)	6 (31.6%)	0.763
Yes	10 (66.7%)	19 (73.1%)		16 (72.7%)	13 (68.4%)	
Tumor number						
Single	11 (73.3%)	21 (80.8%)	0.580	18 (81.8%)	14 (73.7%)	0.530
Multiple	4 (26.7%)	5 (19.2%)		4 (18.2%)	5 (26.3%)	
Tumor size (cm)						
< 5	10 (66.7%)	8 (30.8%)	**0.026**	11 (50.0%)	7 (36.8%)	0.397
≥ 5	5 (33.3%)	18 (69.2%)		11 (50.0%)	12 (63.2%)	
Tumor capsule						
No	7 (58.3%)	17 (77.3%)	0.247	13 (76.5%)	11 (64.7%)	0.452
Yes	5 (41.7%)	5 (22.7%)		4 (23.5%)	6 (35.3%)	
MVI						
No	10 (66.7%)	9 (34.6%)	**0.047**	10 (45.5%)	9 (47.4%)	0.902
Yes	5 (33.3%)	17 (65.4%)		12 (54.5%)	10 (52.6%)	
Tumor differentiation						
I/II	12 (80.0%)	8 (30.8%)	**0.002**	11 (50.0%)	9 (47.4%)	0.867
III/IV	3 (20.0%)	18 (69.2%)		11 (50.0%)	10 (52.6%)	
Adjuvant treatment						
No	7 (46.7%)	6 (23.1%)	0.118	6 (27.3%)	7 (36.8%)	0.511
Yes	8 (53.3%)	20 (76.9%)		16 (72.7%)	12 (63.2%)	
BCLC						
0/A	13 (86.7%)	18 (69.2%)	0.210	16 (72.7%)	15 (78.9%)	0.644
B	2 (13.3%)	8 (30.8%)		6 (27.3%)	4 (21.1%)	
Extrahepatic metastasis						
No	15 (100.0%)	23 (88.5%)	0.172	20 (90.9%)	18 (94.7%)	0.639
Yes	0 (0.0%)	3 (11.5%)		2 (9.1%)	1 (5.3%)	
Intrahepatic recurrence						
No	10 (76.9%)	22 (78.6%)	**0.041**	15 (68.2%)	17 (89.4%)	**0.042**
Yes	3 (23.1%)	6 (21.4%)		7 (31.8%)	2 (10.6%)	

The bold values indicates *P* < 0.05.

### 3.2 Gene mutations and ctDNA analyses

**Fig. 1:**
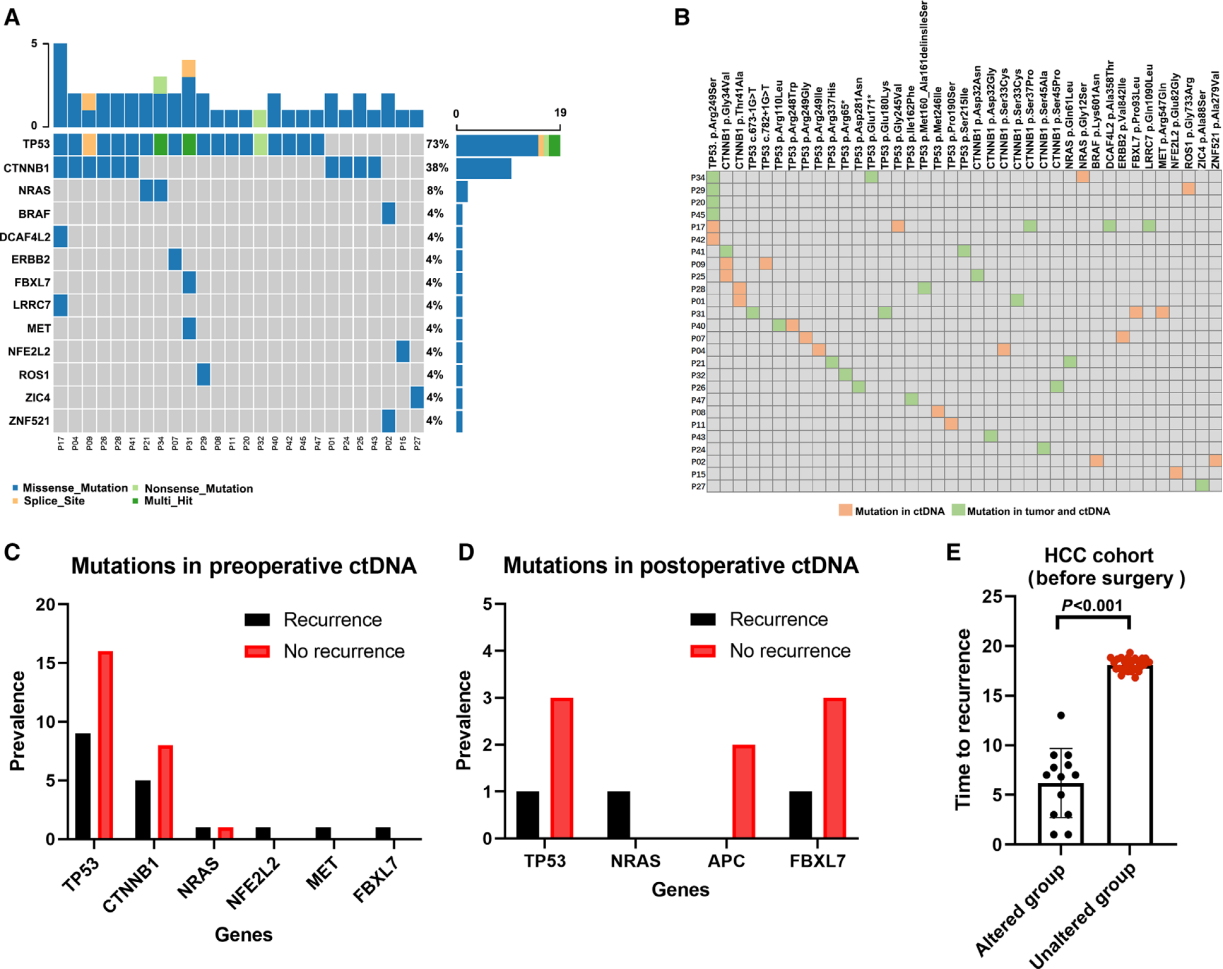
Mutation landscapes and the associations with prognosis. (A) Mutational landscape of 41 patients with HCC, showing a number of somatic mutations in each patient (top), the mutation frequency of each gene (right). (B) Comparison of the ctDNA and HCC tissue mutation profiles, showing the mutation information of each gene (top) and each patient number (left). (C) The mutational discrepancies between recurrence (*n* = 9) and nonrecurrence (*n* = 32) before surgery. Test for comparison of two groups is done by the Wilcoxon test. (D) The mutational discrepancies between recurrence (*n* = 9) and nonrecurrence (*n* = 32) after surgery. Test for comparison of two groups is done by the Wilcoxon test. (E) The association between NRAS, MET, and NEF2L2 mutation and tumor recurrence. *n* = 13 for the altered group and *n* = 28 for the unaltered group. Test for comparison of two groups is done by the Wilcoxon test. The error bars indicate median and SD values.

Figure 1A reveals the ctDNA mutations detected among our HCC group. We identified a total of 47 mutations in the 26 patients and none detected for the remaining patients, with a median of 2 (range, 1–4) gene variations detected in HCC patient. As previously covered, mutations in vital driver genes, such as TP53, CTNNB1, NRAS, BRAF, FBXL7, NFE2L2, and MET, were all clarified with remarkable frequency in this cohort. We then further compared the gene mutation frequencies of driver genes in this HCC cohort from other two public databases, the TCGA datasets and Memorial Sloan Kettering Cancer Center (Fig. S1). According to the captured NGS analyses, the spectrum of genetic mutation in our HCC cohort was highly in concordance with that of two public databases (Fig. S1). Additionally, TP53, CTNNB1, and NRAS were the top three genes detected in our HCC cohort.

In this HCC cohort, we observed a significant association between ctDNA levels and clinical parameters (Fig. S2). Specifically, there was a significant relationship between preoperative cfDNA levels and MVI (*P* = 0.03; 30.99 ng·mL^−1^ compared with 15.7 ng·mL^−1^; Fig. S2), extrahepatic metastases (*P* = 0.02; 23.6 ng·mL^−1^ compared with 14.7 ng·mL^−1^; Fig. S2), or intrahepatic recurrence (*P* = 0.03; 29.4 ng·mL^−1^ compared with 15.3 ng·mL^−1^). However, there were no significant associations between postoperative cfDNA levels (at 1 week) and the presence of MVI, extrahepatic metastases, or intrahepatic recurrence (Fig. S2). Specifically, it was well described that postoperative ctDNA could reflect the low‐residual tumor burden after the curative treatment that should be directly progressed to the recurrence [21]; although our study showed no significant correlation between postoperative cfDNA (detected at 1 week after surgery), other time‐points at 1 month and 4 months in this study revealed that there were significant associations at 4 months between postoperative cfDNA levels (*P* = 0.02) and recurrence (Fig. S3), indicating that postoperative ctDNA may reflect the low‐residual tumor burden after the curative treatment.

Table 2 reveals that a preoperative ctDNA positivity rate was 63.4%, which dropped rapidly to 46% after surgical resection. Statistical analyses showed that the preoperative ctDNA detection rate was also significantly correlated to tumor recurrence (*P* = 0.04), tumor size (*P* = 0.026), MVI (*P* = 0.047), and tumor differentiation (*P* = 0.002). In addition, postoperative ctDNA was only significantly associated with tumor recurrence (*P* = 0.042) but was not associated with other clinicopathologic parameters, including age, sex, tumor differentiation, or tumor size.

Importantly, our results revealed that the positive rate of preoperative ctDNA was notably lower in HCC patients with tumor differentiation I/II (*P* = 0.002), MVI (absent; *P* = 0.047), and smaller tumor size (< 5 cm; *P* = 0.026) than in their counterparts (Table 2).

### 3.3 Mutations in preoperative or postoperative ctDNA and matched HCC tissue

We then investigated the consistence between genetic alterations confirmed in preoperative ctDNA and in matched tumor tissues in our cohort. Forty‐seven gene mutations were detected in ctDNA, and 299 gene mutations were identified in DNA from HCC tissues, of which 25 gene mutations were identified, along with evidence of consistent carcinogenic gene mutations in the matched ctDNA (Fig. 1B, Fig. S4). Six patients had mutations in both the CTNNB1 and TP53 (Fig. 1) genes. Additionally, 23 gene mutations were unique to ctDNA. Through the whole HCC cohort of patients, TP53 (19 of 41 patients, 46.3%), followed by CTNNB1 (10, 24.4%) and NRAS (2, 4.9%; Fig. 1), was the most conventional mutation gene in ctDNA from our HCC cases. Nine of 26 HCC patients with detectable mutations in ctDNA (34.6%) had mutations in the Wnt/β‐catenin signaling pathway, and three (11.5%) had mutations in the RAF/MEK/ERK signaling pathway (Fig. 1B). Missense single nucleotide polymorphism (SNP) mutations (44, 89.8%) were the most of genetic alterations in ctDNA from HCC patients. Notably, patient cases detected by most recurrently mutated genes were changed most after surgery (Fig. S5) was TP53 (16 cases), followed by CTNNB1 (12 cases), NPAP1 (two cases), DCAF4L2 (two cases), and NRAS (one case).

However, to determine the driver mutations contributing to tumor recurrence preoperatively, we explored the clinical distribution of the gene mutations identified in preoperative or postoperative ctDNA based on tumor recurrence status, as shown in Fig. 1C,D. Through comparing the prevalence of gene mutations in these two groups, the mutation rates of NRAS, MET, and NFE2L2 were higher in the recurrence subgroup than in the recurrence‐free subgroup when considering preoperative and postoperative ctDNA mutations, indicating that those gene mutations might be predictors for HCC early intrahepatic recurrence (Fig. 1, Table S1). Interestingly, further analysis noted that patients harboring NRAS, MET, and NFE2L2 mutations had a significantly shorter TTR than patients with unaltered genes (*P* < 0.001; Fig. 1E). Additionally, to investigate the prediction performance of those gene mutations before surgery (NRAS, MET, and NF2E2L2), we used machine learning methods to select predictive factors to predict early recurrence, which showed that the c‐index for median VAF of NRAS, NEF2L2, and MET mutations was 0.80 (Fig. S6A), while the c‐index for combined NRAS, NEF2L2, MET mutations, and clinical parameters (BCLC stage, tumor size, and MVI) was 0.97 (Table S1; Fig. S6B).

### 3.4 The associations between preoperative ctDNA parameters and early recurrence

**Fig. 2:**
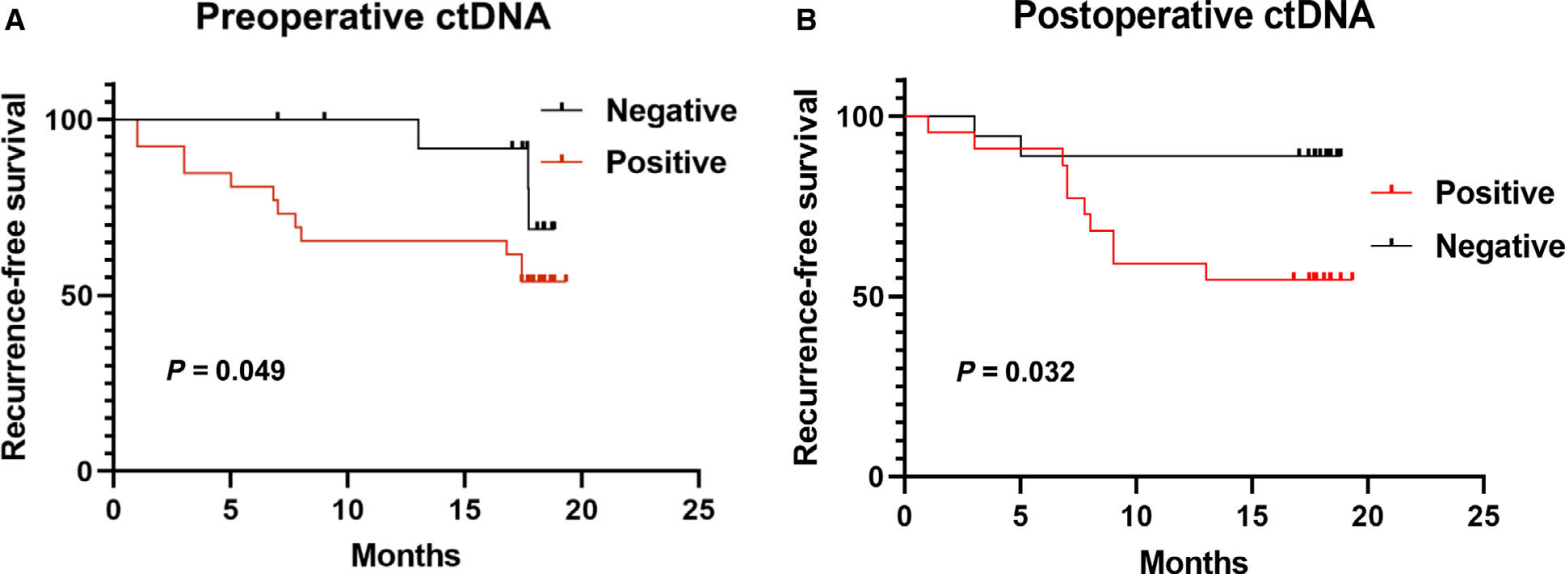
Positivity of ctDNA across two time‐points was significantly associated with tumor recurrence. Preoperative ctDNA (A; *n* = 15 for the negative group and *n* = 26 for the positive group) and postoperative ctDNA (B; *n* = 22 for the positive group and *n* = 19 for the negative group). RFS was estimated using the Kaplan–Meier method, described with median and 95% CI, and compared using the log‐rank test.

**Fig. 3:**
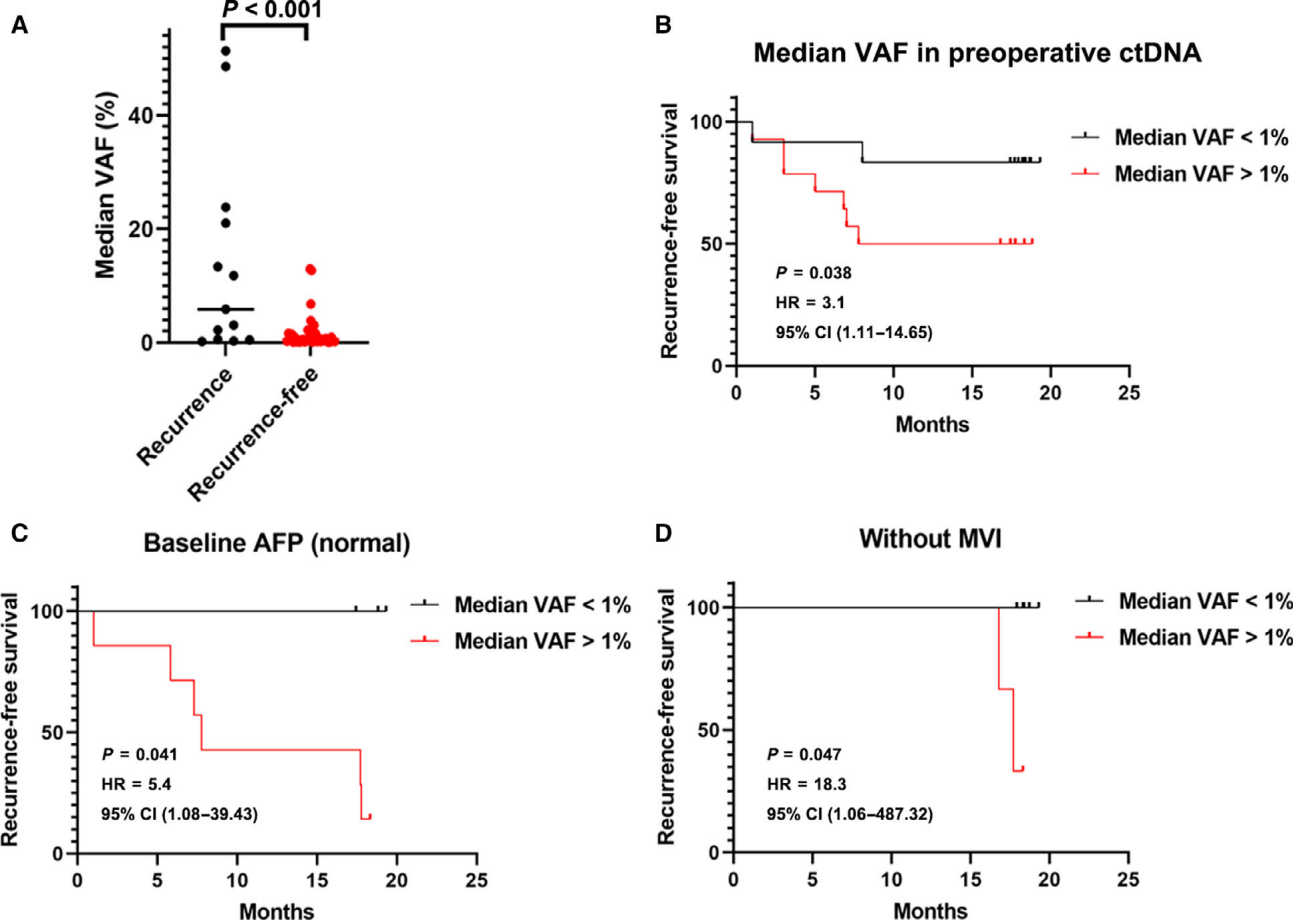
Associations between mutational median VAF of baseline ctDNA with tumor recurrence and clinical parameters. (A) The median VAF distribution of preoperative ctDNA in patients with different recurrence status (*n* = 13 for the recurrence group and *n* = 28 for the recurrence‐free group). Test for comparison of two groups is done by the Wilcoxon test. (B) The Kaplan–Meier analysis of median VAF of preoperative ctDNA (*n* = 12 for median VAF< 1% group and *n* = 13 for median VAF > 1% group). (C) The Kaplan–Meier analysis of median VAF of preoperative ctDNA in patients with normal baseline AFP levels (*n* = 7 for median VAF < 1% group and *n* = 7 for median VAF > 1% group). (D) The Kaplan–Meier analysis of median VAF of preoperative ctDNA in patients with no MVI (*n* = 5 for median VAF < 1% group and *n* = 5 for median VAF > 1% group). VAF at 1% cutoff threshold; RFS was estimated using the Kaplan–Meier method, described with median and 95% CI, and compared using the log‐rank test.

With a median follow‐up of 17.7 months (range, 2.1–19.3), nine patients (Table S2) were found to have early recurrence (all TTR < 2 years) after surgery. The Kaplan–Meier analysis revealed that HCC patients with detectable ctDNA preoperatively were likely to suffer early recurrence in a shorter time than those without detectable ctDNA preoperatively by adjusting BCLC staging (Fig. 2, *P* < 0.05). Similarly, these associations were maintained at other time‐points. An increasing trend over these two time‐points was shown by HRs (preoperative, 2.4; postoperative, 4.3; Fig. 2). When quantifying the ctDNA mutation frequencies, a significant association between the median VAF of mutations in ctDNA and early recurrence was also seen at the two time‐points (Fig. 3B; Fig. S7A). Additionally, the median VAF of baseline ctDNA > 1% demonstrated a powerful predictive performance for early tumor recurrence before treatment (HR, 3.1; *P* = 0.038; Fig. 3A,B), while for common serum tumor markers and tumor recurrence predictors, we also noticed the significant correlations between elevated preoperative AFP (HR, 4.9; *P* = 0.049) or the presence of MVI (HR, 6.9; *P* = 0.036) and early recurrence (Fig. S7B,C). Among the HCC patients with normal preoperative AFP levels or without MVI, however, the median VAF in baseline ctDNA > 1% maintained good predictive performance for early tumor recurrence (HR, 5.4; *P* = 0.041 for the AFP normal group and HR, 18.3; *P* = 0.047 for non‐MVI group, respectively; Fig. 3C,D).

Based on the univariate analysis, preoperative parameters significantly correlated with RFS included the median VAF of mutations in preoperative ctDNA (*P* = 0.01), larger tumor size (> 5 cm, *P* = 0.021), the presence of MVI (*P* = 0.014), the presence of metastasis (*P* = 0.003), and higher BCLC stage (*P* = 0.001). In the multivariate Cox analyses, only the median VAF of mutations in preoperative ctDNA and the presence of MVI remained independent predictors of RFS (HR, 16.5; *P* = 0.036 and HR, 9.0; *P* = 0.016, respectively; Table 3).

**Table 3:** Univariate and multivariate analyses of the association with tumor early recurrence using pretreatment variables.

Variables	Univariate analysis		Multivariate analysis	
	HR (95% CI)	*P* value	HR (95% CI)	*P* value
**Median VAF in preoperative ctDNA (%)**	**60.7 (2.7, 1354.6)**	**0.010**	**16.5 (1.2, 226.6)**	**0.036**
Sex (male vs female)	2.1 (0.3, 15.9)	0.485		
Age, years (≥ 50 vs < 50)	0.4 (0.1, 1.2)	0.096		
HBsAg (positive vs negative)	2.3 (0.5, 10.3)	0.284		
Anti‐HCV treatment (yes vs no)	1.0 (0.1, 8.1)	0.964		
ALT, U·L^−1^ (≥ 75 vs < 75)	1.7 (0.4, 7.8)	0.477		
AFP, ng·mL^−1^ (≥ 20 vs < 20)	1.9 (0.6, 5.5)	0.265		
GGT, U·L^−1^ (≥ 54 vs < 54)	2.2 (0.7, 7.2)	0.183		
Liver cirrhosis (yes vs no)	1.3 (0.4, 4.8)	0.684		
Tumor number (multiple vs single)	1.1 (0.3, 3.9)	0.919		
**Tumor size, cm (> 5 vs ≤ 5)**	**5.9 (1.3, 26.7)**	**0.021**		
Tumor capsule (complete vs incomplete)	1.8 (0.6, 5.5)	0.304		
**Vascular invasion (yes vs no)**	**6.6 (1.5, 30.1)**	**0.014**	**9.0 (1.5, 53.2)**	**0.016**
Tumor differentiation (III/IV vs I/II)	1.8 (0.6, 5.6)	0.295		
Adjuvant treatment (yes vs no)	3.0 (0.7, 13.8)	0.148		
**BCLC (B vs 0/A)**	**6.3 (2.1, 19.2)**	**0.001**		

The bold values indicates *P* < 0.05.

## 4 Discussion

ctDNA has the potential value to revolutionize the clinical practice of multiple cancers, including HCC, by eliminating the necessity for invasive tissue biopsy and conferring dynamic insight into the cancer genomic mutational progression in real time so as to monitor tumor recurrence and prognosis [5, 10, 11, 13]. Nevertheless, compared with other cancers, ctDNA mutation detection in HCC still has some limitations [7, 10, 12]. Despite extensive research, conventional clinicopathologic characteristics have been already considered as predictors of tumor recurrence, including histologic grade, tumor size, clinical stage, and the presence of MVI [2]. Genomic features are deemed to hold huge potential value in predicting tumor recurrence. However, nowadays, there are still a few related researches, and there is a lack of consistence between the results of cohort studies due to the variable methodologies and gene panels utilized. Further explorations are still warranted.

In this report, we applied targeted NGS to investigate the HCC genetic alterations, and the serial changes in ctDNA parameters during surgical therapy for HCC patients. To evaluate the underlying value of predictors for early tumor recurrence, we analyzed clinical factors consisting of the tumor size, tumor number, tumor grade, BCLC staging, serum AFP level, ctDNA detection status, and tumor mutation profile. The results revealed that there were remarkable associations between preoperative cfDNA levels and the presence of MVI, metastases, or recurrence, which is consistent with some studies [9, 10, 11, 13, 14, 22, 23].

Also, we verified the value of ctDNA as a ‘liquid biopsy’ for HCC by showing that genetic alterations were identified in ctDNA according to mutations existing in the matched HCC tissue in our cohort, a vital result that helps to advance this technique further toward the clinical practice. We detected gene mutations shown to be important carcinogenic gene drivers in HCC that have been well verified in the HCC studies, namely, CTNNB1, TP53, NRAS, BRAF, and NFE2L2 [9, 14, 22, 23], which were tested in ctDNA tissues by applying sequencing techniques that utilize comparison‐matched PBMC DNA but not matched HCC DNA to call gene alterations with high accuracy, indicating the applications of this technique in the clinical practice, in which HCC tissue is seldom obtained [24].

Additionally, through analyzing the notable mutated genes in this HCC group, we discovered that harboring NRAS, MET, and NFE2L2 mutations was significantly associated with early recurrence. Previous studies have shown that MET [6, 25], encoded by the HGFR gene, NRAS [26, 27], a known RAS family oncoprotein, and NFE2L2 [28, 29] promote tumor growth and metastasis in HCC, and their somatic mutation can cause liver carcinogenesis [7, 8, 14, 27, 30]. To confirm this result, we obtained gene mutation data from TCGA and found that the gene mutation group had early tumor recurrence; however, this needs to be proven with further studies.

Consistent with the previous studies [10, 11, 22], ctDNA was detectable in up to 63.4% of HCC patients before surgery. After radical liver surgery, the ctDNA positivity rate decreased rapidly to 46%, which might reflect the effect of surgery and that most tumors circulate much less ctDNA in the bloodstream. The above discoveries indicate that, as a marker with a half‐life of < 2 h, serial ctDNA can well reflect dynamic changes in the tumor bulk in real time during the treatment of surgery. Consistent with previous reports [10, 11, 22], our study showed a significant association between preoperative ctDNA and tumor size, tumor differentiation, MVI, and early tumor recurrence (Table 2). However, the lack of relationships between the preoperative ctDNA detection status, the median VAF of preoperative ctDNA, and the treatment response might mirror the reality that tumors have intrinsically variable treatment sensitivities.

Disease progression with early recurrence was observed in nine of the 41 patients with a median follow‐up of 17.7 months. Significant correlations between recurrence and ctDNA detection were observed both preoperatively and postoperatively. Before liver surgery, ctDNA detection correlated with a higher risk of tumor recurrence. This finding confirmed the value of ctDNA in guarding disease progression. Continued ctDNA positivity during surgery could screen out some patients who may experience later tumor recurrence. Moreover, the postoperative time‐point seems to be the most indicative time‐point. At this time‐point, the ctDNA detection in most patients seems to become negative, while those with sustained positive results demonstrated a remarkable high risk of tumor recurrence. Compared with ctDNA detection, higher sensitivity can be seen in the median VAF of preoperative ctDNA.

A high VAF of ctDNA at diagnosis might be reflective of tumor recurrence. With the ability to predict therapeutic failure such an early period, ctDNA might accurately classify the tumor stage, refining regime selection, dynamically adapting the treatment plans, and scheming surgical resection and postoperative therapy. However, these ways sustained to be verified in a randomized clinical setting.

Serum AFP is a common serum biomarker in HCC prognosis; however, the practice of using it to predict patient prognosis is constrained by its modest sensitivity. In this HCC cohort with mainly early‐stage tumors, the abnormal rate of baseline AFP was merely 36.5%. For patients with normal baseline AFP values, based on the median VAF of preoperative ctDNA, their RFS curves can still be remarkably distinguished. Compared with AFP, ctDNA was a more sensitive and accurate tumor marker, which can be used to monitor tumor burden and predict patient prognosis.

Our data verified the previous reports that ctDNA has good prognostic performance for predicting the patient survival [10, 11, 31]. Here, we showed that the preoperative status of ctDNA for the first time can predict tumor recurrence before the initiation of surgery. This significant correlation was also observed in previous studies on ctDNA analyses [17, 32] but at later postoperative time‐points. This high sensitivity may be due to the large panel size of our parallel targeted genes and the sequencing depth of our application in NGS analysis, which makes it possible to detect more low‐frequency mutations, although at this stage, such analysis requires a lot of laboratory work and relatively high cost.

This study also has some limitations, which mainly consist of small sample size and relatively short follow‐up time. However, the strong statistical power (HR, 3.3; *P* = 0.032) of the relationships between the median VAF of preoperative ctDNA and RFS in this report, along with the previous findings from other reports [14, 33, 34], gives prominence to the prognostic advantages of ctDNA in HCC patients during the treatment of surgery. We reported the value of preoperative ctDNA for the first time in predicting early tumor recurrence in patients with HCC. We are waiting for the future results of this ongoing study to validate above findings and clarify the value of ctDNA for long‐term results. Future studies need a larger cohort and long‐term follow‐up to clarify these related findings.

## 5 Conclusion

Our results suggest that ctDNA might be a precise dynamic tumor biomarker, which can reflect the tumor burden of HCC patients in real time. We proved that ctDNA detection and VAF detection can be used as prognostic factors for early recurrence before treatment. In addition, we observed that NRAS, MET, and NFE2L2 mutations may predict early tumor recurrence. In conclusion, series ctDNA analysis combined with tumor tissue genomic sequencing can confer precise information for predicting and monitoring tumor recurrence, and help to optimize individualized multimodal treatment strategies. Based on our findings, more researches are warranted to further verify these findings.

## Conflict of interest

The authors declare that there is no conflict of interest that could be perceived as prejudicing the impartiality of the research reported.

## Author contributions

G‐QZ, W‐RL, ZT, RH, H‐XS, W‐FQ, X‐FJ, C‐YT, JZ, and Y‐HS designed the study. G‐QZ, W‐RL, RH, S‐SS, and C‐YT collected and prepared patient tissues. W‐RL, RH, ZD, and YF did the statistical analyses. RH, HW, and C‐YT prepared figures. G‐QZ, W‐RL, Z‐BD, Y‐FP, RH, C‐YT, JZ, YF, JG, JF, and Y‐HS reviewed the results, interpreted data, and wrote the manuscript. All authors have made an intellectual contribution to the manuscript and approved the submission.

## Ethics approval and consent to participate

The present study was performed in accordance with the 1975 Declaration of Helsinki. Approval for the use of human subjects was obtained from the Research Ethical Committee of Zhongshan Hospital, and informed consent was obtained from each individual enrolled in this study.

### Peer Review

The peer review history for this article is available at https://publons.com/publon/10.1002/1878‐0261.13105.

## Supporting information


**Fig. S1**. The mutational frequency against two publicly available datasets in the context of recurrent driver genes.
**Fig. S2.** Comparison of cfDNA levels between clinical parameters in patients with hepatocellular carcinoma (HCC).
**Fig. S3.** Comparison of cfDNA levels between recurrence or non‐recurrence in patients with hepatocellular carcinoma (HCC) at different time points (at 1 month, *n* = 5 for no recurrence group and *n* = 9 for recurrence group; At 4 months, *n* = 3 for no recurrence group and *n* = 5 for recurrence group).
**Fig. S4.** The mutation profile of ctDNA and hepatocellular carcinoma (HCC) tissue DNA, showing mutated genes in each patients (left), shared mutations both in ctDNA and HCC tissue (red spot).
**Fig. S5.** Prevalence by genes in patients with hepatocellular carcinoma (HCC) before and after surgery.
**Fig. S6.** Prediction performance of ctDNA and clinical parameters by receiver operating characteristic curve (ROC) analysis.
**Fig. S7.** The Kaplan–Meier analysis of median variant allele frequency (VAF) of ctDNA for tumor recurrence.
**Table. S1.** Predictive factors associated with hepatocellular carcinoma (HCC) early recurrence by using machine learning method.
**Table. S2.** Gene variants detected in preoperative or postoperative ctDNA from early recurrence hepatocellular carcinoma (HCC) patients.

## Data Availability

The data are not available in a public database or repository. The datasets generated and analyzed in this study are available from the corresponding author on reasonable request.
