# Supplementary material for: Serial circulating tumor DNA to predict early recurrence in patients with hepatocellular carcinoma: a prospective study
Source: Mol Oncol. 2021 Oct 4;16(2):549–61. doi: 10.1002/1878-0261.13105 (PMC8763657; doi:10.1002/1878-0261.13105)
Supplement: Supplementary file 1 — Fig. S1. The mutational frequency against two publicly available datasets in the context of recurrent driver genes. Fig. S2. Comparison of cfDNA levels between clinical parameters in patients with hepatocellular carcinoma (HCC). Fig. S3. Comparison of cfDNA levels between recurrence or non‐recurrence in patients with hepatocellular carcinoma (HCC) at different time points (at 1 month, n = 5 for no recurrence group and n = 9 for recurrence group; At 4 months, n = 3 for no recurrence group and n = 5 for recurrence group). Fig. S4. The mutation profile of ctDNA and hepatocellular carcinoma (HCC) tissue DNA, showing mutated genes in each patients (left), shared mutations both in ctDNA and HCC tissue (red spot). Fig. S5. Prevalence by genes in patients with hepatocellular carcinoma (HCC) before and after surgery. Fig. S6. Prediction performance of ctDNA and clinical parameters by receiver operating characteristic curve (ROC) analysis. Fig. S7. The Kaplan–Meier analysis of median variant allele frequency (VAF) of ctDNA for tumor recurrence. Table. S1. Predictive factors associated with hepatocellular carcinoma (HCC) early recurrence by using machine learning method. Table. S2. Gene variants detected in preoperative or postoperative ctDNA from early recurrence hepatocellular carcinoma (HCC) patients. [file MOL2-16-549-s001.docx]

**Supplementary Material**

**
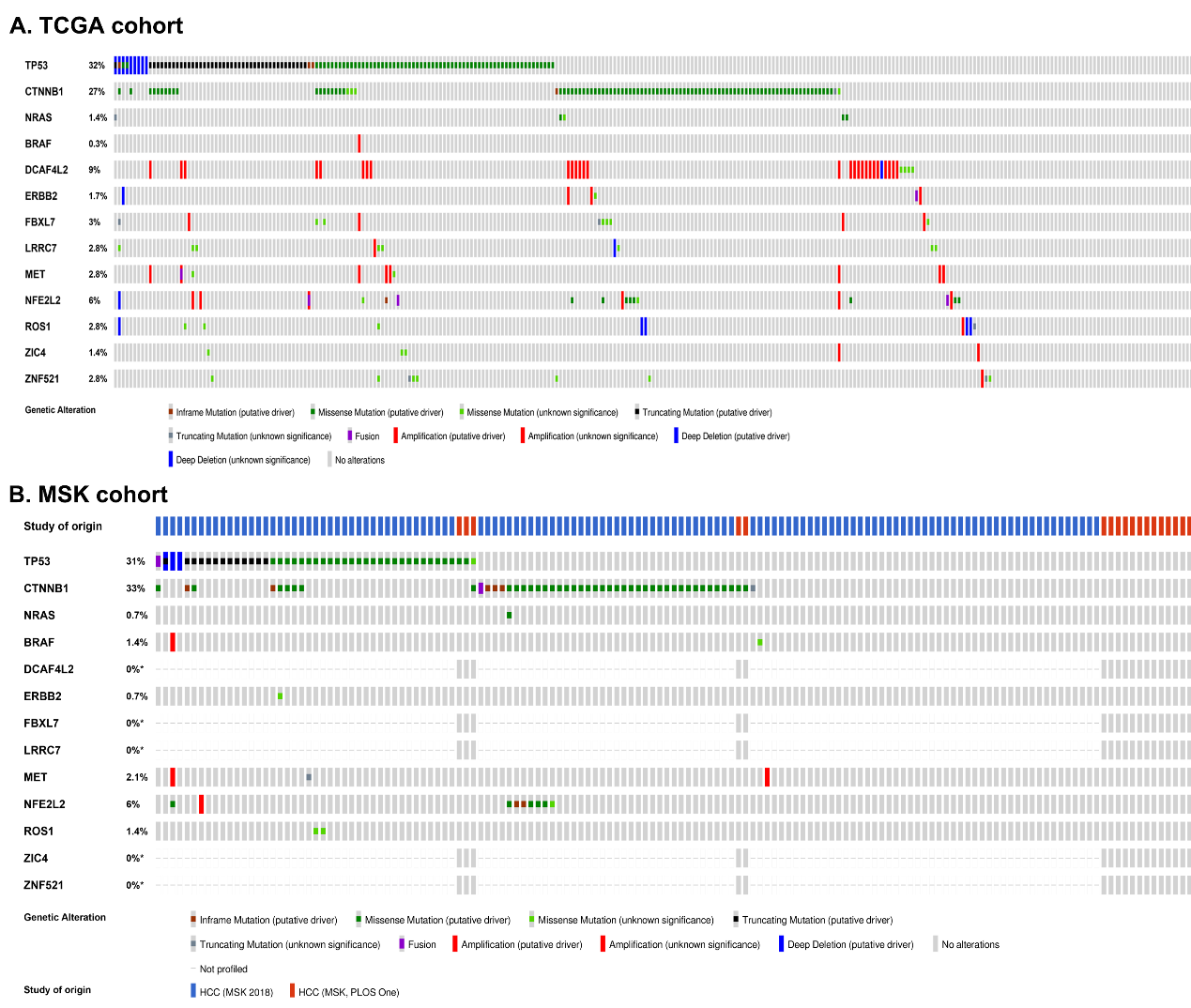
**

**figure S1.** The mutational frequency against two publicly available datasets in the context of recurrent driver genes. TCGA cohort (A), MSK cohort (B).

**

**

**figure S2.** Comparison of cfDNA levels between clinical parameters in patients with hepatocellular carcinoma (HCC). A. n=38 for no metastasis group; n=3 for metastasis group. B. n=32 for no recurrence group; n=9 for recurrence group. C. n=27 for no microvascular invasion (MVI) group; n=14 for MVI group. D. n=38 for no metastasis group; n=3 for metastasis group. E. n=32 for no recurrence group; n=8 for recurrence group. C. n=19 for no microvascular invasion (MVI) group; n=22 for MVI group.

**
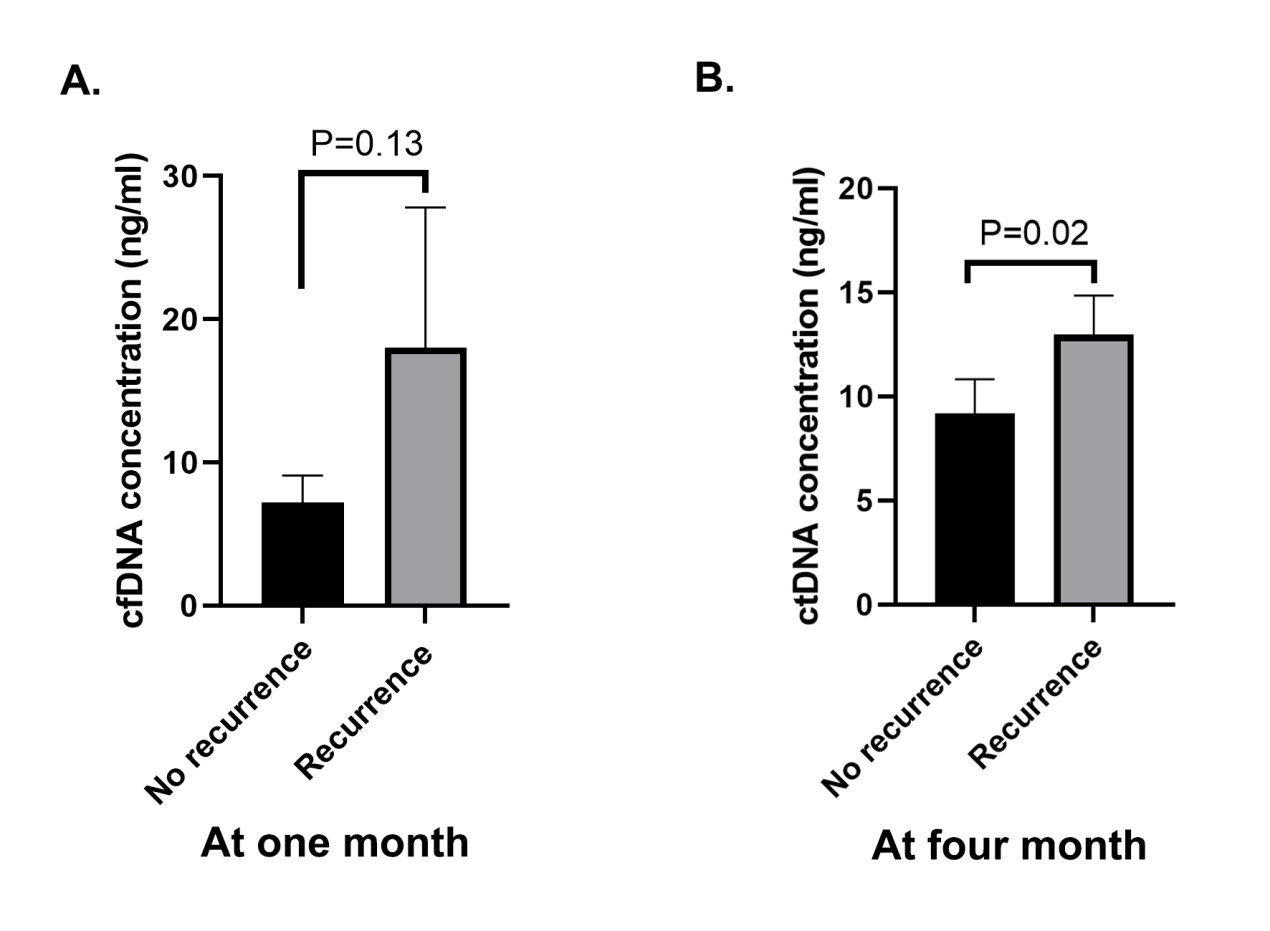
**

**figure S3.** Comparison of cfDNA levels between recurrence or non-recurrence in patients with hepatocellular carcinoma (HCC) at different time points (at one month, n=5 for no recurrence group and n=9 for recurrence group; At four months, n=3 for no recurrence group and n=5 for recurrence group).

**
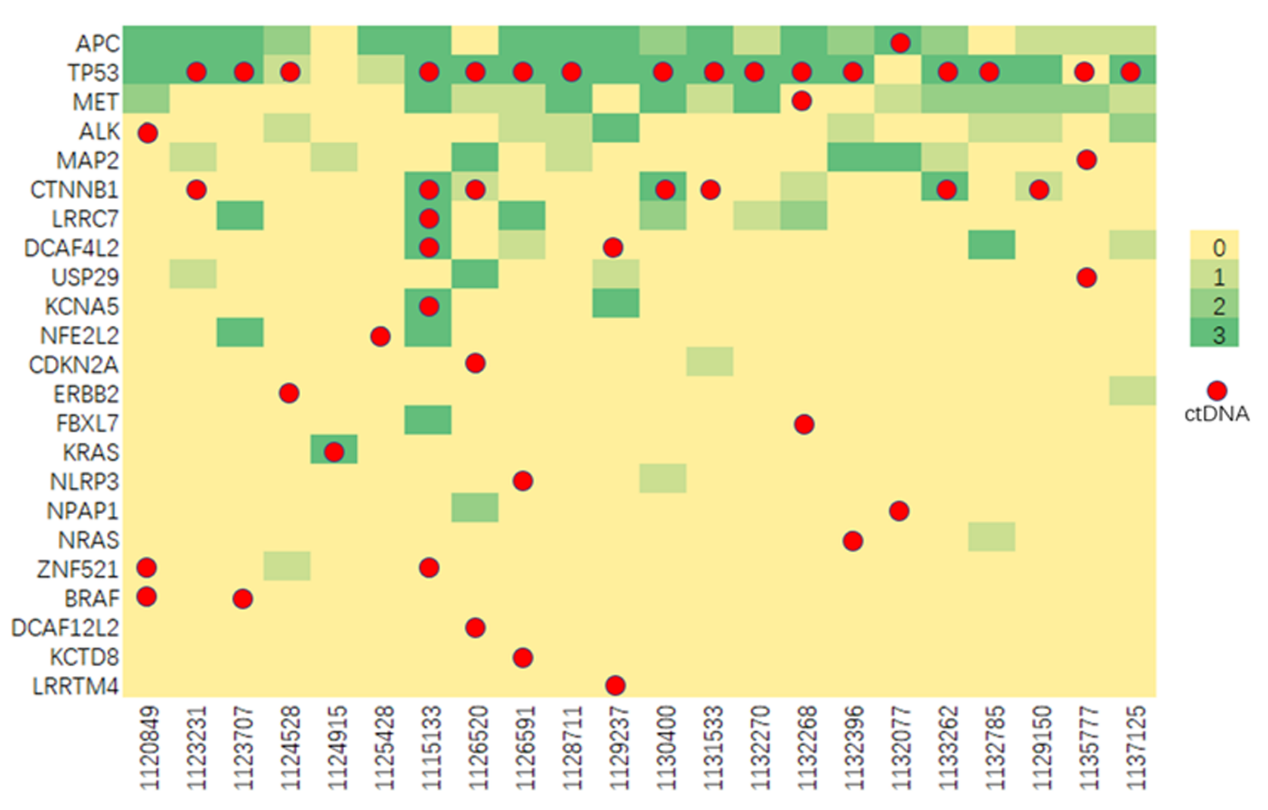
**

**figure S4.** The mutation profile of ctDNA and hepatocellular carcinoma (HCC) tissue DNA, showing mutated genes in each patients (left), shared mutations both in ctDNA and HCC tissue (red spot).

**
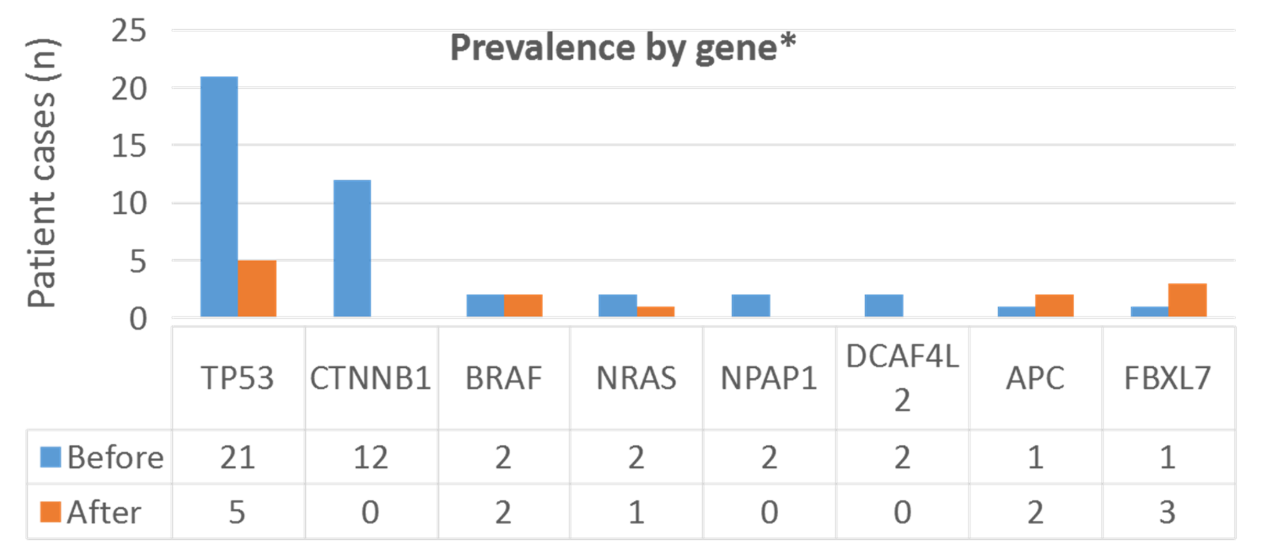
**

**figure S5.** Prevalence by genes in patients with hepatocellular carcinoma (HCC) before and after surgery.

**
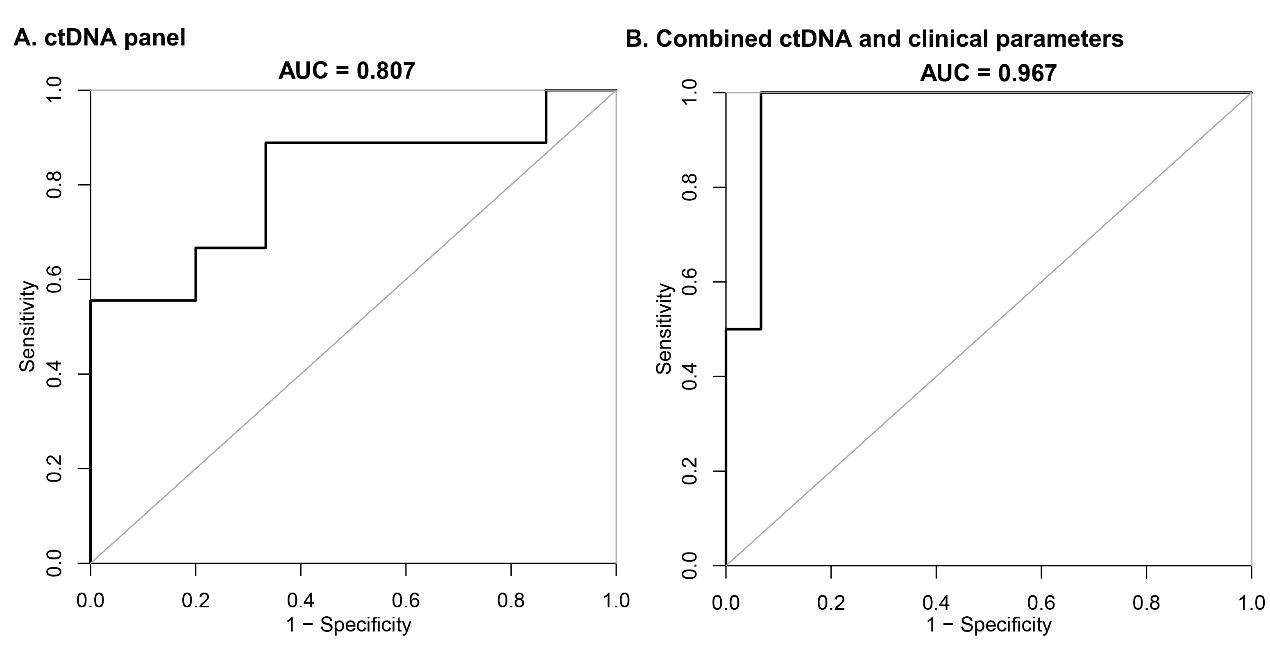
**

**figure S6.** Prediction performance of ctDNA and clinical parameters by receiver operating characteristic curve (ROC) analysis. A, The ROC analysis of NRAS, MET and NEF2L2 mutations detected in ctDNA. B, The ROC analysis of ctDNA gene panel and clinical parameters.

**
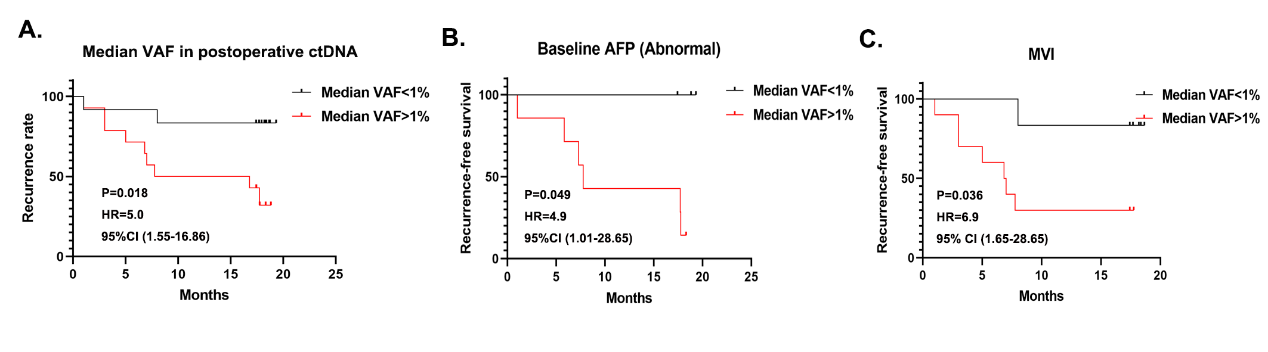
**

**figure S7.** The Kaplan-Meier analysis of median variant allele frequency (VAF) of ctDNA for tumor recurrence. A, The Kaplan-Meier analysis of median VAF of postoperative ctDNA (n=12 for median VAF<1% group and n=13 for median VAF>1% group). B, The Kaplan-Meier analysis of median VAF of preoperative ctDNA in patients with abnormal α-fetoprotein (AFP) levels (n=6 for median VAF<1% group and n=7 for median VAF>1% group). C, The Kaplan–Meier analysis of median VAF of preoperative ctDNA in patients with microvascular invasion (MVI; n=6 for median VAF<1% group and n=9 for median VAF>1% group).

**table S1.** Predictive factors associated with hepatocellular carcinoma (HCC) early recurrence by using machine learning method.

| Variable | Coefficients | Overall permuted P-value |
| --- | --- | --- |
| **Preoperative median VAF of NRAS, NEF2L2, MET mutations** | 0.236 | **0.0267** |
| Preoperative ctDNA |  |  |
| Negative | - | 0.653 |
| Positive | - | 0.653 |
| **BCLC** |  |  |
| 0-A | -0.361 | **<0.0001** |
| B | 0.283 | **<0.0001** |
| Tumor number |  |  |
| Single |  | 0.413 |
| Multiple |  | 0.413 |
| **Tumor size (cm)** |  |  |
| <5 |  | **0.0267** |
| >5 |  | **0.0267** |
| Tumor differentiation |  |  |
| I-II |  | 0.253 |
| II-III |  | 0.253 |
| AFP (U/L) |  |  |
| <20 |  | 0.153 |
| >20 | 0.017 | 0.153 |
| **Microvascular invasion** |  |  |
| No | -0.499 | **<0.0001** |
| Yes |  | **<0.0001** |
| Prediction performance | | |
| C-index for median VAF of NRAS, NEF2L2, MET mutations | 0.80 |  |
| C-index for combined parameters | 0.97 |  |

**table S2.** Gene variants detected in preoperative or postoperative ctDNA from early recurrence hepatocellular carcinoma (HCC) patients.

| **Patient number** | **Treatment period** | **Gene** | **Coding Change** | **Amino Acid Change** | **Variant Description** | **Allele Fraction** | **Time to recurrence (m)** |
| --- | --- | --- | --- | --- | --- | --- | --- |
| P43 | Preoperative | CTNNB1 | c.95A>G | p.Asp32Gly | Missense variant | 0.1178 | 6.83 |
| P34 | Preoperative | TP53 | c.747G>T | p.Arg249Ser | Missense variant | 0.1338 | 5 |
| P34 | Preoperative | TP53 | c.511G>T | p.Glu171* | Stop gained | 0.0588 | 5 |
| P34 | Preoperative | NRAS | c.34G>A | p.Gly12Ser | Missense variant | 0.0013 | 5 |
| P08 | Preoperative | TP53 | c.738G>T | p.Met246Ile | Missense variant | 0.2381 | 3 |
| P01 | Preoperative | CTNNB1 | c.121A>G | p.Thr41Ala | Missense variant | 0.0048 | 1 |
| P01 | Preoperative | CTNNB1 | c.110C>G | p.Ser37Cys | Missense variant | 0.0022 | 1 |
| P15 | Preoperative | NFE2L2 | c.245A>G | p.Glu82Gly | Missense variant | 0.0019 | 8 |
| P32 | Preoperative | TP53 | c.193A>T | p.Arg65* | Stop gained | 0.2101 | 1 |
| P31 | Preoperative | TP53 | c.538G>A | p.Glu180Lys | Missense variant | 0.0308 | 3 |
| P31 | Preoperative | TP53 | c.673-1_673delinsTT | N/A | Splice acceptor variant & Intron variant | 0.0222 | 3 |
| P31 | Preoperative | FBXL7 | c.278C>T | p.Pro93Leu | Missense variant | 0.0032 | 3 |
| P31 | Preoperative | MET | c.1640G>A | p.Arg547Gln | Missense variant | 0.001 | 3 |
| P45 | Preoperative | TP53 | c.747G>T | p.Arg249Ser | Missense variant | 0.4857 | 7.77 |
| P28 | Preoperative | TP53 | c.480_481delinsTT | p.M160_A161delinsIS | Missense variant | 0.513 | 7 |
| P28 | Preoperative | CTNNB1 | c.121A>G | p.Thr41Ala | Missense variant | 0.0057 | 7 |
| P34 | Postoperative | NRAS | c.34G>A | p.Gly12Ser | Missense variant | 0.0025 | 5 |
| P34 | Postoperative | FBXW7 | c.1514G>A | p.Arg505His | Missense variant | 0.0018 | 5 |
| P31 | Postoperative | TP53 | c.673-1_673delinsTT | N/A | Splice acceptor variant & Intron variant | 0.0019 | 3 |
| P31 | Postoperative | ALK | c.1169A>G | p.Gln390Arg | Missense variant | 0.0011 | 3 |
